# Anesthetic Management of a Cesarean Delivery in a Patient With MYH11 Mutation Who Underwent Aortic Replacement

**DOI:** 10.7759/cureus.48391

**Published:** 2023-11-06

**Authors:** Takashi Kawasaki, Michiko Sugita, Chisato Kodera, Naoyuki Hirata

**Affiliations:** 1 Department of Anesthesiology, Kumamoto University Hospital, Kumamoto, JPN; 2 Department of Obstetrics and Gynecology, Kumamoto University Hospital, Kumamoto, JPN

**Keywords:** genetic testing, combined spinal-epidural anesthesia, familial thoracic aortic aneurysm and dissection, myh11, cesarean delivery

## Abstract

We report a case of a pregnant woman with a history of ascending arch replacement for aortic dissection who still had a residual descending aortic dissection. She underwent urgent genetic testing to identify hereditary aortic-related diseases that might be useful in perinatal management. A mutation in the myosin heavy chain gene (MYH11), indicating a high risk of aortic dissection but a low impact on other vascular systems and organs, was identified. Due to concerns about the development of residual aortic dissection, cesarean delivery with combined spinal-epidural anesthesia was selected. Predelivery genetic testing might be useful for perinatal anesthetic management.

## Introduction

Pregnancy is associated with a significantly increased risk of aortic dissection, with an incidence that is 4-25-fold higher than that in the nonpregnant state [1,2]. In high-risk pregnant women, delivery and anesthetic management should be considered based on the mother’s general diagnosis. Hereditary aortic disease has different effects on each organ depending on its causative gene, so genetic testing before delivery can be useful in perinatal management. In this case, genetic testing revealed abnormalities in the myosin heavy chain gene (MYH11), and perinatal management was discussed with the expert cardio-obstetric team.

## Case presentation

**Figure 1:**
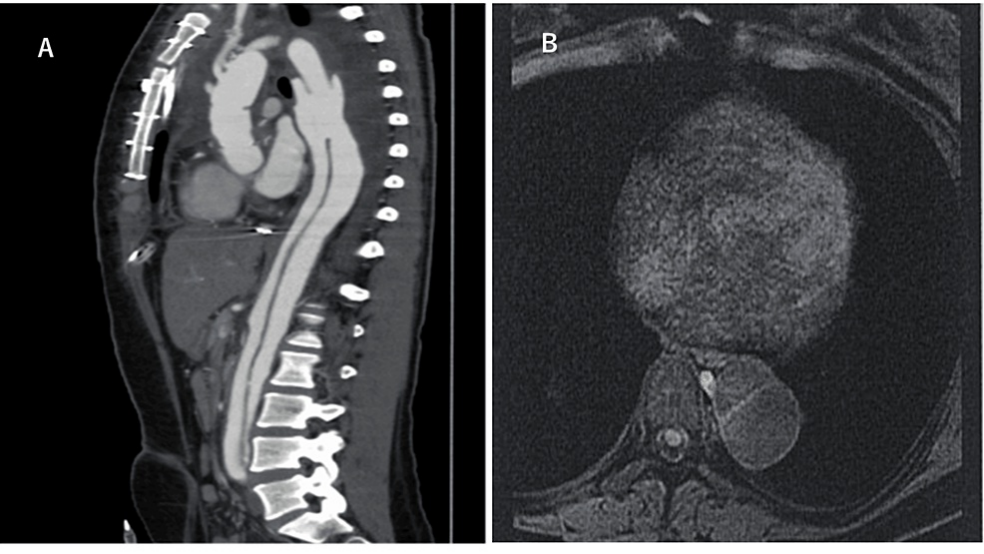
Contrast computed tomography image and MRI T2-weighted image A: Contrast computed tomography image taken before pregnancy shows residual aortic dissection from the distal arch aorta to just above the common iliac artery bifurcation. B: MRI T2-weighted image taken at 37 weeks' gestation shows a residual dissected cavity in the distal arch of the replacement, but no diameter enlargement.

The patient was a 28-year-old woman (height, 165 cm; weight, 73.5 kg; gravida 2, para 0). At 22 years of age, she had aortic dissection from the ascending to the descending aorta and subsequently underwent ascending and aortic arch replacement. Postoperatively, the residual dissection remained below the descending aorta just above the bifurcation of the common iliac artery (the maximum diameter was 40 mm) (Figure 1), and the risk of rupture was not considered high. She took beta-blockers for the first six months after aortic replacement; however, the medication was discontinued due to hypotension. She had no family history of aortic disease, and her physical examination results were normal.

She conceived spontaneously, and in the second trimester, her systolic blood pressure increased to approximately 120 mmHg. Her cardiologists considered pregnancy safe to continue; however, the development of descending aortic dissection was concerning, and blood pressure control was recommended. The patient was administered 750 mg/day of methyldopa at 36 weeks of gestation and then switched to 20 mg/day of nifedipine at 37 weeks of gestation, and the dose was increased to 40 mg/day to control blood pressure.

Considering that the identification of hereditary thoracic aortic disease could be useful in perinatal management, genetic testing was urgently performed at 35 weeks of gestation. She carried a variant in MYH11 (c.4578+1G>A), leading to a diagnosis of nonsyndromic aortic dissection, with a high risk of aortic dissection but with little impact on other vasculature or organs. MYH11 mutation has also been associated with patent ductus arteriosus (PDA), but she was not diagnosed. After consultation with the expert cardio-obstetric team, elective cesarean delivery was scheduled at 37 weeks of gestation under combined spinal-epidural anesthesia. We set the goal for perinatal management of systolic blood pressure to <120 mmHg.

A radial artery line was placed for continuous hemodynamic monitoring, and two peripheral intravenous (IV) lines were placed. Her pre-anesthetic blood pressure was 135/85 mmHg, and continuous administration of nicardipine (0.2-0.5 μg/kg/min) was initiated. An epidural catheter was placed at the T12-L1 interspace and then spinal anesthesia was performed at the L3-L4 interspace using a 25G Quincke needle. A total of 11.5 mg of isobaric bupivacaine and 10 μg of fentanyl were administered intrathecally. Due to inadequate anesthesia levels, 5 mL of 2% lidocaine was administered into the epidural space. Fortunately, a sufficient anesthetic level was obtained by avoiding hemodynamic changes. The infant was born with an Apgar score of 9/9 at 1/5 min, and the umbilical artery blood pH was 7.364. Five units of oxytocin were intravenously administered immediately after delivery, and favorable uterine contractions were obtained. During the operation, the blood pressure increased gradually; therefore, nicardipine administration was restarted at 0.7 μg/kg/min and continued until after the operation. Postoperative pain was controlled by continuous epidural administration (0.2% ropivacaine mixed with fentanyl). IV nicardipine administration was continued until postoperative day 6 and then switched to oral administration. The patient was discharged on postoperative day 10 without any complications.

## Discussion

**Figure 2:**
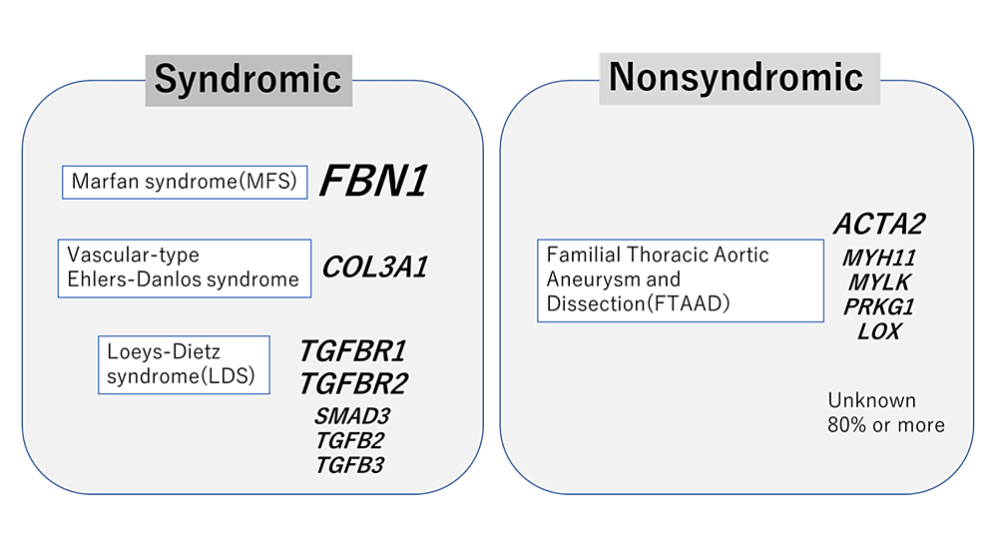
Hereditary thoracic aortic aneurysms and dissections and major causal genes Hereditary thoracic aortic aneurysms and dissections are distinguished between syndromic and nonsyndromic, each involving a number of genes [4]. Image: Author's own creation.

Pregnancy has been noted as an important risk factor for acute aortic dissection. Estrogen increases the degeneration of collagen and elastin in the intima of the aortic wall and promotes aortic remodeling. Additionally, estrogen increases hemodynamic stress on the weakened aortic wall during the third trimester of pregnancy [3]. Familial thoracic aortic aneurysm and dissection (FTAAD) is a hereditary nonsyndromic aortic disease caused by mutations in several genes (MYH11, ACTA2, and MYLK) that are associated with aortic wall integrity, and the phenotype is exclusively a cardiovascular abnormality. Conversely, Marfan syndrome, Ehlers-Danlos syndrome, and Loeys-Dietz syndrome (LDS) are syndromic and affect multiple organ systems (Figure 2) [4]. Therefore, prior genetic testing to identify genetic mutations may be useful for risk assessment for perinatal management. In Marfan syndrome and LDS, spinal anesthesia may be ineffective because of the uneven distribution of anesthetic agents caused by dural dilation [5]. In LDS cases, uterine rupture has been reported; hence, it is preferable to achieve early delivery and avoid high abdominal pressure by performing cesarean delivery with preparation for massive hemorrhage [5]. In our case, a mutation was found in MYH11, which encodes a component of smooth muscle contractile proteins, namely, the coiled-coil region of the smooth muscle myosin heavy chain. Loss of MYH11 affects the formation of myosin filaments, resulting in decreased vascular smooth muscle and mucoid degeneration. Reportedly, MYH11-deficient patients have reduced aortic compliance, higher pulse wave velocity, and an increased risk of aortic dissection and PDA than healthy individuals [6]. Crawford et al. reported on the perinatal management of a pregnant woman with an MYH11 mutation. In that case, LDS was initially suspected based on the clinical symptoms. Cesarean delivery was performed at 27 weeks of gestation before receiving subsequent ascending aortic arch replacement, and the MYH11 mutation was identified postoperatively [7]. In our case, we opted for cesarean delivery to prevent the progression of residual descending aortic dissection. The reason for this is that perinatal descending aortic dissection after aortic arch replacement has been previously reported [8,9]. Genetic testing prior to delivery has enabled safer perinatal management. To our knowledge, this is the second case report on the perinatal management of a pregnant woman with an MYH11 mutation.

## Conclusions

We performed a cesarean delivery on a pregnant patient who underwent ascending arch aortic replacement and had residual dissection with a mutation in the MYH11 gene. Genetic testing before delivery might be useful for determining perinatal management in a high-risk pregnant woman with a history of aortic dissection.
